# Gallium Liquid Metal Nanoparticles as Agents to Treat Multidrug‐Resistant Bacterial Infections

**DOI:** 10.1002/mbo3.70078

**Published:** 2025-10-23

**Authors:** Hang Thi Nguyen, Jiratchaya Puangseree, Tien Thanh Nguyen, Manh Tuong Nguyen, Christopher Leigh, Henrietta Venter, Krasimir Vasilev, Rungtip Chuanchuen, Darren J. Trott, Vi Khanh Truong, Abiodun David Ogunniyi

**Affiliations:** ^1^ Australian Centre for Antimicrobial Resistance Ecology, School of Animal and Veterinary Sciences The University of Adelaide Roseworthy South Australia Australia; ^2^ ARC Training Centre for Environmental and Agricultural Solutions to Antimicrobial Resistance The University of Queensland Brisbane Queensland Australia; ^3^ Research Unit for Microbial Food Safety and Antimicrobial Resistance, Department of Veterinary Public Health, Faculty of Veterinary Science Chulalongkorn University Bangkok Thailand; ^4^ Biomedical Nanoengineering Laboratory, College of Medicine and Public Health Flinders University Bedford Park South Australia Australia; ^5^ Adelaide Microscopy The University of Adelaide Adelaide South Australia Australia; ^6^ Health and Biomedical Innovation, Clinical and Health Sciences University of South Australia Adelaide South Australia Australia

**Keywords:** biofilms, gallium liquid metal nanoparticles, antimicrobial resistance, scanning electron microscopy, superbugs, transmission electron microscopy

## Abstract

Multidrug‐resistant (MDR) bacterial infections constitute one of the top global public health threats. This study investigated the potential of gallium liquid metal nanoparticles (GaLM NPs) as a new agent against MDR pathogens. GaLM NPs was bactericidal against methicillin‐resistant *Staphylococcus aureus* (MRSA) isolates and a vancomycin‐intermediate *S. aureus* reference strain (minimum inhibitory concentration (MIC) and minimum bactericidal concentration (MBC) values between 39 and 156 μg/mL). The bactericidal activity of GaLM NPs was supported by transmission electron microscopy showing marked ultrastructural changes in the cell envelope of MRSA USA300. GaLM NPs were bacteriostatic against selected Gram‐negative (*Acinetobacter baumannii*, *Pseudomonas aeruginosa* and *Klebsiella pneumoniae*) reference strains and isolates (MICs between 39 and 625 μg/mL). Furthermore, GaLM NPs demonstrated additive and bacteriostatic activity when combined with sub‐inhibitory concentrations of colistin against *P. aeruginosa* isolates. Additionally, GaLM NPs showed anti‐biofilm activity against MRSA USA300 (minimum biofilm eradication concentration of 625 μg/mL); morphology changes of GaLM NPs‐treated cells was demonstrated by scanning electron microscopy. Finally, GaLM NPs was safe to human epidermal keratinocyte cell line at 1024 µg/mL (6.5 × MIC). We conclude that GaLM NPs warrant further exploration for the effective treatment of Gram‐positive or Gram‐negative infections both alone and in combination with antimicrobial drugs.

## Graphical abstract

Gallium liquid metal nanoparticles (GaLM NPs) are promising agents for treating multidrug‐resistant Gram‐positive and Gram‐negative bacterial infections.(A), Preparation of GaLM NPs suspension; (B, C), Morphology of GaLM NPs without and with F127, scale bar: 500 nm; (D), Diameter distribution of GaLM NPs; (E), TEM showing effects of GaLM NPs treatment on morphology of MRSA USA300; (F), SEM showing eradication of MRSA USA 300 biofilms by GaLM NPs.

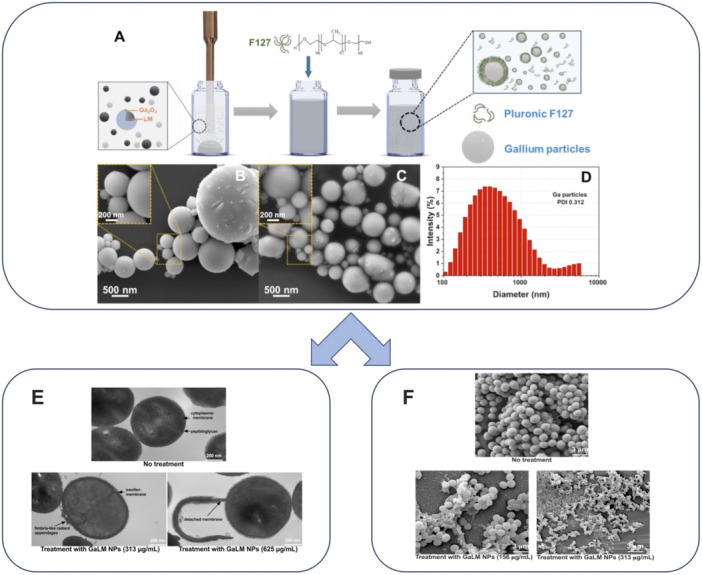

## 1 Introduction

Bacterial pathogens, particularly those that are resistant to multiple drug classes, continue to be responsible for serious infections, causing massive global morbidity and mortality. The 2019 Lancet Global Burden of Disease Study indicates that bacterial infections are the second leading cause of global deaths (behind ischemic heart disease), accounting for 7.7 million (1 in 8) deaths (Antimicrobial Resistance Collaborators 2022a). Leading drug‐resistant bacterial pathogens were directly responsible for 929,000 deaths and were associated > 3.5 million deaths due to antimicrobial resistance (AMR) (Antimicrobial Resistance Collaborators 2022b). AMR‐related deaths are projected to increase to > 10 million/year by 2050 if not addressed, with cumulative economic impact rising to over 100 trillion USD (O'Neill 2016). *Staphylococcus aureus* was the leading bacterial cause of death in 135 countries and was also associated with the most global deaths in individuals > 15 years. Thus, there is an urgent clinical need to develop new antimicrobial agents with novel chemistry and mechanisms of action, which also prevent cross‐resistance to existing drug classes.

To address this pressing need, current literature indicates that combination therapy, development of new drug classes, phage therapy and antibiotic resistance breakers such as novel efflux pump inhibitors are critically important new approaches for treating multidrug‐resistant pathogens. However, there are only approximately 60 antibiotics in the clinical development pipeline to date, and the vast majority of these are derivatives of existing antibiotic classes designed to overcome some of the known resistance mechanisms (Butler et al. 2022; Theuretzbacher 2023; Paterson 2024; Theuretzbacher et al. 2020a; Theuretzbacher et al. 2020b). Furthermore, the rate of approval of new drugs by the Food and Drug Administration for multidrug‐resistant pathogens is not in line with their increasing significance (Pew Charitable Trusts 2022). Given the problems surrounding clinical development and licensure of new antibiotics, alternative approaches are being actively pursued. For instance, the notable antibacterial properties of silver have been known for decades (Bruna et al. 2021). However, emerging reports of bacteria exhibiting resistance to silver raise concerns about the long‐term efficacy of silver‐based antimicrobial treatments (Hosny et al. 2019). This rapidly evolving landscape highlights the need for more research into alternatives or complementary strategies to combat the threat of silver‐resistant bacterial infections. In this context, gallium (Ga) is emerging as a viable alternative metal for various medical applications due to its unique properties and advantages.

Ga in ionic forms (Ga^3+^) has been shown to exhibit moderate activity against a wide range of pathogens, positioning it as a versatile solution for addressing bacterial infections (Kircheva and Dudev 2020; Choi et al. 2024; Li et al. 2022). For instance, gallium nitrate (Ga(NO_3_)_3_) showed in vivo efficacy against *A. baumannii* in a *Galleria mellonella* larvae model (Antunes et al. 2012). Notably, Ga in salt forms also exhibits biocompatibility; for example, Ga‐nitrate and Ga‐citrate are known to promote bone growth (Yu et al. 2020). Ga^3+^ possesses a unique mechanism of action by disrupting bacterial iron metabolism, thereby lowering the propensity for bacteria to develop resistance (Goss et al. 2018). Also, gallium liquid metal nanoparticles (GaLM NPs) have been shown to possess antimicrobial properties, and were found to strongly interact with microbial cells (Cheeseman et al. 2022a). GaLM NPs have been found to elicit an anti‐inflammatory response without interfering with the ferric homeostasis pathway in immune cells (Zhang et al. 2022). Ga metal has the intriguing ability to alloy with other antimicrobial metals, forming distinctive combinations that enhance both antimicrobial efficacy and osseointegration properties (Nguyen et al. 2023). However, to our knowledge, a detailed and systematic examination of the activity of GaLM NPs against multidrug‐resistant bacterial infections is yet to be reported in the literature. Therefore, in this study, we examined in vitro activity of GaLM NPs alone or in combination with antibiotics for the treatment of multidrug‐resistant infections.

## 2 Materials and Methods

### 2.1 Antibiotics and Chemicals

Colistin sulfate and vancomycin hydrochloride were purchased from Sigma‐Aldrich (Australia). Stock solutions containing 25.6 mg/mL of each compound (dissolved in water) were stored in 1 mL aliquots at −20°C; DMSO (Thermo Fisher Scientific, Australia). Ruthenium red and L‐lysine acetate were purchased from Sigma‐Aldrich (Australia) and dissolved into the working concentrations. Fixatives (4.0% paraformaldehyde, 1.25% glutaraldehyde, CaCl_2_ 0.01 M, 4% sucrose and 0.075% ruthenium red, 0.075% L‐lysine acetate in 0.1 M cacodylate buffer); phosphate‐buffered saline (PBS), ethanol and sucrose (Chem‐supply, Australia); osmium tetroxide and hexamethydisilazane (HDMI) (ProScitech, Australia) were provided by Adelaide Microscopy, The University of Adelaide (Adelaide, SA, Australia).

### 2.2 Synthesis and Characterization of GaLM NPs

**Figure 1:**
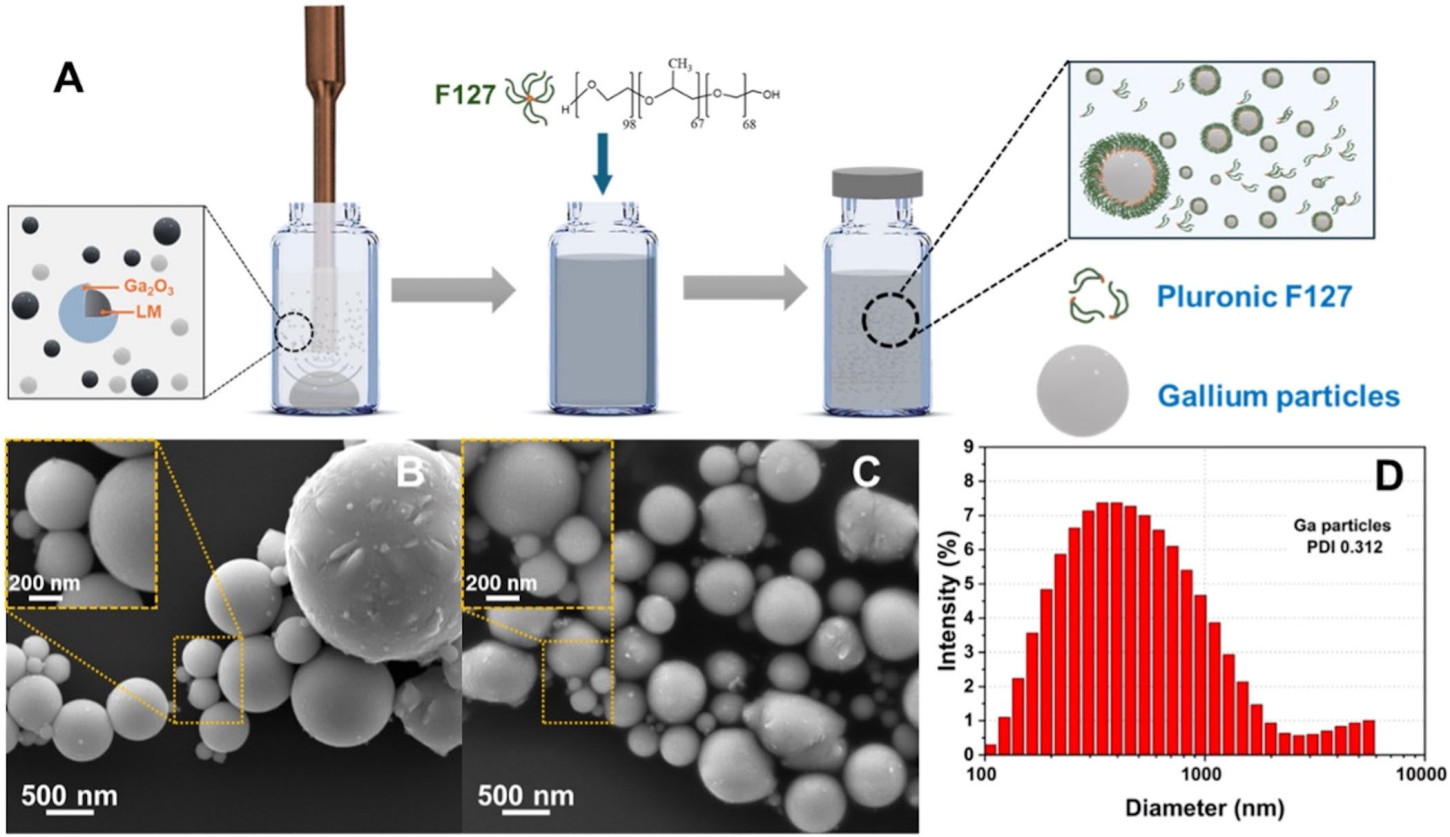
Preparation and characterization of GaLM NPs. (A) A schematic showing the preparation process of GaLM NPs suspension. (B, C) The morphology of GaLM NPs without and with F‐127, scale bar: 500 nm. (D) The graph shows the diameter distribution of prepared GaLM NPs.

The synthesis of GaLM NPs was conducted using methods outlined in our earlier studies (Nguyen et al. 2023; Truong et al. 2023; Lin et al. 2017). Briefly, approximately 150 mg of solid Ga was added to 9 mL of Milli‐Q water. It was then subjected to sonication for 40 min using a standard probe sonicator (Vibra‐Cell, Sonics) set up within an ice bath to yield a suspension of Ga particles. The sonication was conducted in an ice bath to mitigate the heat‐induced formation of crystalline gallium oxyhydroxide (GaOOH) with a rod‐shaped structure (Lin et al. 2017; Lin et al. 2018). Thereafter, 1 mL of 10% (w/v) Pluronic F‐127 solution (Sigma‐Aldrich) was added into 9 mL of GaLM NPs suspension as a surfactant to ensure the Ga suspension's stability, preventing rapid sedimentation, followed by mixing with a vortex mixer (Figure 1A). F‐127 is a FDA‐approved nontoxic polymer notable for its biomedical application viability (Gioffredi et al. 2016), and known for features such as low toxicity, reversible temperature‐sensitive gelation, drug encapsulation capability and gel formation at low concentrations (Brunet‐Maheu et al. 2009; Rahdar et al. 2022; Khattak et al. 2005). The surface morphology of the GaLM NPs was investigated via SEM on a FEI Inspect F50 instrument. Before the imaging, GaLM NPs were washed twice with Milli‐Q water to remove the F127 and subsequently left to dry at room temperature. The GaLM NPs were imaged at 10 kV. The size of the Ga particles was determined at room temperature using dynamic light scattering with a ZetaSizer Nano ZS (Malvern Instruments, Malvern, UK).

### 2.3 Bacterial Strains and Growth Conditions

Clinical MRSA isolates (*n* = 20) were obtained from the Australian Centre of Antimicrobial Resistance Ecology (ACARE) collection. Bioluminescent MRSA USA300 (SAP231) and community‐acquired MRSA MW2 (SAP227) (Plaut et al. 2013) were a kind gift from Roger Plaut (Food and Drug Administration, Bethesda, MD, USA); vancomycin‐intermediate *S. aureus* (VISA; ATCC 700699) was provided by College of Medicine and Public Health, Flinders University. MSSA (ATCC 29213 and ATCC 49775), MRSA ATCC 43300, *A. baumannii* (NCIMB 12457 and ATCC 19606), *P. aeruginosa* (ATCC 27853 and PAO1), and *K. pneumoniae* ATCC 13883 were provided by SA Pathology (Adelaide, SA, Australia). Clinical *P. aeruginosa* isolates (*n* = 22) were obtained from Flinders Medical Centre (Bedford Park, SA, Australia). Bioluminescent *Escherichia coli* Xen14 (derived from the parental strain *E. coli* WS2572) and bioluminescent *P. aeruginosa* Xen41 (derived from the parental strain PAO1) were purchased from PerkinElmer Inc. (Hopkinton, MA, USA). All bacteria were stored at ‐80°C in Luria Bertani (LB) broth (Edwards, Australia) with 20% glycerol in a PC2 microbiology laboratory on level 4 Basil Hetzel Building, University of South Australia.

All bacterial identities were confirmed by matrix‐assisted laser desorption/ionization time‐of‐flight mass spectroscopy (MALDI‐TOF/MS) (microflex LT/SH Biotyper; Bruker Daltonik, Leipzig, Germany) at ACARE before antimicrobial susceptibility testing. Bacteria were grown overnight on horse blood agar (HBA) and in LB broth. For selection, MRSA USA300 (SAP231) was grown on HBA containing 10 μg/mL chloramphenicol, *E. coli* Xen14 was grown on HBA containing 30 μg/mL kanamycin and *P. aeruginosa* Xen41 was grown on HBA containing 60 μg/mL tetracycline.

### 2.4 Antimicrobial Susceptibility Testing

Antimicrobial activity of GaLM NPs was identified by minimum inhibitory concentration (MIC), which is the lowest concentration of drug that prevents the growth of a microorganism after incubation (CLSI 2010).

The MICs of GaLM NPs and antibiotics against MSSA (ATCC 29213 and ATCC 49775), MRSA reference and clinical isolates, VISA (ATCC 700699), *A. baumannii* (NCIMB 12457 and ATCC 19606), *E. coli* (WS2572 [Xen14]), *P. aeruginosa* reference and clinical isolates, and *K. pneumoniae* (ATCC 13883), were determined in flat‐bottom 96‐well microtiter plates (Sarstedt 82.1581.001, Nümbrecht, Germany), using the modified broth micro‐dilution method recommended by the Clinical and Laboratory Standards Institute (CLSI 2010) with slight modifications. Briefly, bacterial suspensions were prepared in 2 × LB broth at 10^6^ × CFU (colony‐forming unit)/mL from overnight grown HBA plates (2 × sub‐culture) and 50 μL of the suspension dispensed into each well of flat‐bottom 96‐well plates. GaLM NPs were prepared by serial two‐fold dilutions of stock solution (15 mg/mL) in saline. 50 μL of each dilution was then further diluted 1:2 in 50 μL of bacterial suspensions in flat‐bottom 96‐well plates. GaLM NPs concentrations ranged from 5 µg/mL to 5000 µg/mL. Serial two‐fold dilutions of colistin and vancomycin were prepared from stock solutions (25.6 mg/mL) in saline. 50 μL of each dilution was further diluted 1:2 in 50 μL of bacterial suspensions such that vancomycin and colistin concentrations ranged from 0.03 to 256 µg/mL while final bacterial suspensions were 5 × 10^5^ CFU/mL in each well. The negative growth control was LB broth only, and the positive growth control was bacterial suspension in LB broth.

Each MIC test was carried out in duplicate and performed on two separate occasions. The minimum bactericidal concentration (MBC) was recorded as the lowest concentration of each test compound, at which a 99.9% colony count reduction was observed on the plate (Barry et al. 1999).

### 2.5 Synergy Testing by Checkerboard Assay

Potential interaction between GaLM NPs with colistin or vancomycin was determined by a modification of the standard checkerboard assay described previously (Nguyen et al. 2021; Ogunniyi et al. 2017; Pi et al. 2020). Briefly, the antibiotic and GaLM NPs were prepared as described for the MIC testing above. One microliter of each antibiotic solution for each antibiotic‐GaLM NPs combination was dispensed along the abscissa (from row A to F) of the flat‐bottom 96‐well microtiter plates using a 12.5 μL electronic multichannel pipette (VIAFLO Voyager II, Biotools); 50 μL GaLM NPs of each concentration ranged from 5 to 2500 μg/mL was dispensed along the ordinate (from column 3 to column 12) followed by the addition of 50 μL of bacterial suspensions (1 × 10^6^ CFU/mL prepared in 2 × LB) to achieve the final 5 × 10^5^ CFU/mL in each well. The negative growth control was LB broth only, and the positive growth control was bacterial suspension in LB broth.

One plate was used for each strain/isolate, and the plates were incubated at 37°C for 20 h and observed visually; *A*_600 nm_ measurement was not applied due to the black color of GaLM NPs, as shown in Supporting Information S1: Figure S1. The interaction of GaLM NPs and other antibiotics was calculated using the fractional inhibitory concentration index (Hwang et al. 2012) as described previously (Nguyen et al. 2021; Ogunniyi et al. 2017; Pi et al. 2020). The dose reduction index was used to describe the difference between the effective dose of GaLM NPs in combination with colistin or vancomycin from the individual dose of each compound. A typical example of a checkerboard assay of combined GaLM NPs with colistin against *P. aeruginosa* cln17 is depicted in Supporting Information S1: Figure S1.

*Fractional inhibitory concentration index (FICI)*. The interaction of two antibiotics was calculated as the FICI using the following formula:

$$\text{FICI}\;=\frac{{\text{MIC}}_{\text{A in combination}}}{{\text{MIC}}_{\text{A alone}}}+\frac{{\text{MIC}}_{\text{B in combination}}}{{\text{MIC}}_{\text{B alone}}}.$$

A is GaLM NPs and B is colistin or vancomycin. According to FICI, the interaction between two antibiotic agents was interpreted as follows: synergistic (FICI ≤ 0.5); additive or partially synergistic (0.5 < FICI ≤ 1); indifferent (1 < FICI ≤ 4); and antagonistic (FICI > 4) (Hwang et al. 2012).

*Dose reduction index (DRI)*. The DRI was used to describe the difference between the effective doses of a single compound in combination with its individual dose. DRI was calculated using the following formula:

$$\text{DRI}\;=\frac{{\text{MIC}}_{\text{A alone}}}{{\text{MIC}}_{\text{A in combination}}}.$$

A DRI is clinically relevant when the dose reduction is associated with a reduction in toxicity without reducing efficacy. DRI ( > 1) is considered beneficial (Eid et al. 2012).

### 2.6 Time‐ and Concentration‐Dependent Growth Inhibition and Kill‐Kinetics Assays

Time‐ and concentration‐dependent growth inhibition and kill‐kinetic assays of GaLM NPs alone or in combination with colistin or vancomycin were performed (in duplicate) against bioluminescent MRSA USA300 (SAP231), *P. aeruginosa* (Xen41) and *E. coli* (Xen14) essentially as described for MIC and synergy determinations. The assays were performed using 100 μL volumes in black 96‐well microtiter plates with clear bottoms (CLS3603, Sigma‐Aldrich) covered with Breathe‐Easy sealing membrane (Z380059, Sigma‐Aldrich). The plates were incubated for 20 h overnight at 37°C, and bacterial metabolic activity (in relative light units) was measured every 20 min for 18 h on a Cytation 5 Multi‐Mode Reader (BioTek, Winooski, VT, USA). Bioluminescent MRSA USA300 (SAP231), *P. aeruginosa* Xen41 and *E. coli* Xen14 were used in these assays because the optical density (*A*_600 nm)_ of bacteria could not be measured due to the black color of GaLM NPs.

Time‐kill kinetics of GaLM NPs against MRSA USA300 (SAP231), *P. aeruginosa* Xen41 and *E. coli* Xen14 were performed on two separate occasions as described previously (Nguyen et al. 2021). Briefly, a few colonies of MRSA USA300 (SAP231) or *P. aeruginosa* Xen41 grown overnight on HBA were dissolved in normal saline and adjusted to *A*_600 nm_ = 0.1 (equivalent to approx. 1 × 10^8^ CFU per mL), and the bacterial suspension was further diluted 1:100 in 2 × LB broth. GaLM NPs were prepared at concentrations ranging from 1 × MIC to 4 × MIC in saline. Then, 1 mL of bacterial suspension was added with 1 mL of different concentrations of GaLM NPs to obtain 0.5 × MIC to 2 × MIC. Duplicate cultures were incubated at 37°C and 20 μL of samples were withdrawn at 0, 0.5, 1, 2, 4, 6, 8 and 24 h, serially diluted 10‐fold and plated in duplicate on HBA overnight at 37°C for bacterial enumeration. Vancomycin and colistin were used as drug controls for the Gram‐positive and Gram‐negative assays, respectively.

### 2.7 Minimum Biofilm Eradication Concentration (MBEC) Testing

Biofilm activity testing of GaLM NPs and vancomycin against MRSA USA300 (SAP231) was performed using the MBEC^TM^ biofilm assay (MBEC Biofilm Inoculator, Innovotech, Edmonton, Canada). Briefly, MRSA USA300 (SAP231) was grown overnight on HBA containing 10 µg/mL chloramphenicol, subcultured twice, and then a single colony was grown overnight in LB broth supplemented with 10 µg/mL chloramphenicol for selection. Biofilm formation on pegs was established by inoculating the device with 150 µL of 1 × 10^6^ CFU/mL in LB supplemented with 1% glucose per well and placed on a rocking platform (Select BioProducts, Model SBS300‐2, New Jersey, United States) at 37°C at 70 rpm for 24 h, which allows a shear force to be created forming equivalent biofilms on all pegs. Biofilm‐formed pegs were washed twice in PBS for 10 s on each occasion to remove planktonic bacterial cells, then exposed to 200 µL of LB supplemented with 1% glucose containing different concentrations of GaLM NPs and vancomycin in challenge plates prepared as for MIC testing and then incubated at 37°C for another 24 h on a rocking platform at 70 rpm. Following treatment, the biofilms formed on all pegs were washed twice in PBS for 10 s on each occasion. The biofilm‐formed pegs were then transferred to a recovery 96 well‐plate containing 200 µL of fresh LB standing for 30 min at room temperature to equilibrate before being disrupted via sonication (Soniclean, Model 160TD, Australia) at 100 V for 30 min to dislodge the biofilm. Biofilm susceptibility of MRSA USA300 (SAP231) to GaLM NPs and vancomycin was assessed quantitatively by CFU determination to ascertain bacterial viability on each peg. Briefly, 20 μL of samples were withdrawn from each well of recovery 96‐well plates, serially diluted 10‐fold and plated in duplicate on HBA overnight at 37°C for bacterial enumeration. The recovery plate was also incubated at 37°C for 24 h to determine MBEC, defined as the minimum concentration of antimicrobials that eradicate the biofilm evidenced by clear wells.

### 2.8 Transmission Electron Microscopy (TEM)

#### 2.8.1 Preparation of Bacterial Cells

The bioluminescent MRSA USA300 (SAP231) used above was cultured on an HBA plate containing 10 µg/mL chloramphenicol overnight at 37°C in an incubator. A single colony was taken from the overnight growth on HBA and dissolved in 10 mL LB broth containing 10 µg/mL chloramphenicol for selection in a 50 mL flask and incubated at 37°C under continuous agitation in an orbital shaker at 150 rpm. The overnight bacterial culture was diluted 1:30 in 40 mL LB broth in a 50 mL flask of LB broth and then incubated again at 37°C under continuous agitation until *A*_600 nm_ = 0.5. The SAP231 was treated with GaLM NPs at 313 µg/mL and 625 µg/mL for 3 h at 37°C under continuous agitation in a shaker at 150 rpm. During treatment, each sample was manually mixed every 10 min to ensure adequate GaLM NPs contact with the cells. A treatment time of 3 h was determined empirically to be optimal for visualization of the effects of GaLM NPs treatments on the MRSA USA300 (SAP231) cells.

#### 2.8.2 Sample Processing for TEM

The TEM protocol was modified from a previous study (Nguyen et al. 2021). Briefly, treated samples were centrifuged at 123 × *g* for 5 min at 4°C to separate bacterial cells from the GaLM NPs. The untreated and treated cells were then harvested by centrifugation at 2900 × *g* for 5 min at 4°C to avoid cell damage and washed twice in cacodylate buffer supplemented with 4% sucrose. The cells were then fixed in 1 mL fixative containing 4.0% paraformaldehyde, 1.25% glutaraldehyde, CaCl_2_ 0.01 M, 4% sucrose and 0.035% ruthenium red, 0.075% L‐lysine acetate in 0.1 M cacodylate buffer, pH 7, overnight at 4°C. The fixed cells were then washed twice in cacodylate buffer, followed by post‐fixation in 1% osmium tetroxide in cacodylate buffer containing 0.035% ruthenium red for 1 h at room temperature. Subsequently, all samples were washed twice in cacodylate buffer. Then, cells were dehydrated using a graded series of 50%, 70%, 90% and 100% ethanol for 10 min (2 × for each step and 3 × for 15 min in 100% ethanol) at room temperature. Thereafter, the cells were infiltrated in propylene oxide: Epon‐Araldite resin (50:50 ratio). Samples were incubated in 100% Epon‐Araldite resin overnight at room temperature under rotation, followed by one resin change 24 h later. Subsequently, the cells were polymerized in fresh Epon‐Araldite resin at 70°C for 48 h. Sections were cut to 1 μm using a glass knife, stained with 1% toluidine blue containing 1% borax and viewed under a light microscope at 400 × magnification to identify stained bacteria. At least four ultra‐thin sections were then cut to 80 nm with an ultramicrotome (Leica) using a diamond knife (Diatome) and placed on 200‐mesh copper EM grids (Proscitech). Sections were sequentially stained with uranyl acetate (4% in dH_2_O), and Reynolds leads citrate for 10 min each, with three washes in distilled water in between each stain. Sections were then viewed between 25000 × and 130,000 × on a Tecnai G2 Spirit 120 kV Transmission Electron Microscope (FEI Company). Images were obtained at 130,000 × magnification and analyzed using Olympus Soft Imaging Systems.

### 2.9 Scanning Electron Microscopy (SEM)

#### 2.9.1 Sample Processing for SEM

The SEM sample processing protocol was modified from a previous study (Andersson et al. 2021; Abdo et al. 2025). Briefly, the peg lid was prepared as described above for the MBEC assay. After 24 h treatment, the peg lid was washed twice in PBS for 10 s, then fixed in 200 μL fixatives containing 4.0% paraformaldehyde, 1.25% glutaraldehyde and 4% sucrose overnight at 4°C. The fixed cells were washed twice in PBS, followed by post‐fixation in 1% osmium tetroxide in PBS buffer for 1 h at room temperature. Subsequently, all samples were washed twice in PBS. Then, cells were dehydrated using a graded series of 50%, 70%, 90% and 100% ethanol for 10 min (2 × for each step, but 3 × for 15 min in 100% ethanol) at room temperature. Thereafter, the cells were dehydrated once in 100% ethanol: HDMI (50:50) and then twice in 100% HDMI at room temperature. Subsequently, the cells were dried overnight in a biosafety cabinet. Samples were coated with 5 nm platinum, then viewed using SEM (FEI Inspect F50) and imaged at 6 kV.

### 2.10 Mammalian Cell Toxicity Assays

The cytotoxicity of different concentrations (128, 256, 512 and 1024 µg/mL) of GaLM NPs to HaCaT cell line was assessed using quantitative real‐time polymerase chain reaction (PCR) to measure the expression of the proliferation marker gene Ki67, as previously described (Cheeseman et al. 2022b). After 24 h treatment, RNA was extracted from untreated and GaLM NP‐treated HaCaT cells using the easy‐BLUE Total RNA Extraction Kit (iNtRON Biotechnology). Briefly, cDNA was then synthesized using the PrimeScript RT Reagent Kit (Perfect Real Time) (Takara, RR037A) following manufacturer guidelines using primer sequences: Human *ACTB* forward 5′–CAC CAT TGG CAA TGA GCG GTT C–3′ and human *ACTB* reverse 5′–AGG TCT TTG CGG ATG TCC ACG T–3′; human *MKI67* forward: 5'–GAA AGA GTG GCA ACC TGC CTT C–3′ and human *MKI67* reverse 5'–GCA CCA AGT TTT ACT ACA TCT GCC–3′. Quantitative real‐time PCR was run in triplicate using the CFX Opus 384 Real‐Time PCR System (Bio‐Rad). Relative gene expression of *MKI67* was normalized to housekeeping genes (*ACTB*) and calculated by using the comparative ΔΔC_T_ method (Cheeseman et al. 2022b).

## 3 Results

### 3.1 GaLM NPs Synthesis and Characterization

GaLM NPs were synthesized as described in the Materials and Methods section. The schematic in Figure 1A highlights the morphology of GaLM NPs, showing a liquid Ga core encased in native oxide layers of GaOOH (Goff et al. 2021). This distinct structure endows the GaLM NPs with adhesive properties, arising from the synergistic influence of the surrounding oxides and the malleability of the liquid core (Truong et al. 2023; Cheeseman et al. 2022b; Kwon et al. 2021). In addition, the controlled use of the FDA‐approved nontoxic polymer F‐127 surfactant with the added properties of low toxicity, reversible temperature‐sensitive gelation, drug encapsulation capability and gel formation at low concentrations, promotes GaLM NPs stability and prevents rapid sedimentation (Figure 1C).

The particle size distribution analysis (Figure 1D) indicates that prolonging the ultrasonication to 40 min results in smaller and more uniformly sized Ga particles (Nguyen et al. 2023). Hence, the finalized average dimension of the GaLM NPs was determined to be 401.2 ± 8.7 nm at the 40‐min mark, a specification that guided the synthesis of GaLM NPs for subsequent experiments.

### 3.2 GaLM NPs Are Active against Gram‐Positive and Gram‐Negative Bacteria

The antimicrobial activity of GaLM NPs was tested against three *S. aureus* ATCC reference strains, 20 clinical MRSA isolates (Ogunniyi et al. 2017) and one VISA ATCC 700699 listed in Table 1 and Supporting Information S1: Table S1 using vancomycin as a drug control. The GaLM NPs MIC was 78 µg/mL for the MSSA reference strains and 39 µg/mL for VISA ATCC 700699; whereas the MIC range was 78–156 µg/mL for 20 MRSA isolates. The GaLM NPs MBC/MIC ratio was lower than 4, indicating bactericidal activity against *S. aureus* pathogens.

**Table 1:** Antimicrobial activity of GaLM NPs against MSSA and MRSA strains and isolates.

*S. aureus* strains/isolates	MIC (µg/mL)		MBC/MIC		MIC_50_ (µg/mL)		MIC_90_ (µg/mL)	
	GaLM NPs	Van^b^	GaLM NPs	Van	GaLM NPs	Van	GaLM NPs	Van
ATCC 29213	78	1	1	1	—*	—	—	—
ATCC 49775	78	1	1	1	—	—	—	—
ATCC 12600 (Xen29)	78	1	1	1	—	—	—	—
MRSA (*n* = 20)	78‐156	1	1	1	156	1	156	1
VISA ATCC 700699^a^	39	4	1	1	—	—	—	—

Abbreviations: MBC, minimum bactericidal concentration; MIC, minimum inhibitory concentration; MRSA, methicillin‐resistant *S. aureus.*

^a^ VISA (ATCC 700699), vancomycin‐intermediate *S. aureus*.

^b^ Van, vancomycin (used as a drug control). MIC tests were undertaken on three separate occasions; —* = not determined.

GaLM NPs MIC was 625 µg/mLfor two *A. baumannii* reference strains (*A*. ATCC 19606 and NCIMB 12457), 156 µg/mL for *K. pneumoniae* ATCC 13883 and greater than 5000 µg/mL for *E. coli* Xen14. The GaLM NPs MIC_50_ and MIC_90_ values for *P. aeruginosa* ATCC reference strains and clinical isolates were both 78 µg/mL (Table 2).

**Table 2:** Antimicrobial activity of GaLM NPs against Gram‐negative bacterial pathogens.

Bacterial strains	MIC^a^ (µg/mL)		MBC/MIC^b^		MIC_50_ (µg/mL)		MIC_90_ (µg/mL)	
	GaLM NPs	Col^c^	GaLM NPs	Col	GaLM NPs	Col	GaLM NPs	Col
*A. baumannii* ATCC 19606	625	1	> 4	1	—*	—	—	—
*A. baumannii* NCIMB 12457	625	1	> 4	1	—	—	—	—
*K. pneumoniae* ATCC 13883	156	0.25	> 4	1	—	—	—	—
*E. coli* WS2572 (Xen14)	> 5000	0.5	—	1	—	—	—	—
*P. aeruginosa* ATCC 27823	78	1	> 4	2	—	—	—	—
*P. aeruginosa* PAO1 (Xen41)	78	1	> 4	2	—	—	—	—
*P. aeruginosa* (n = 21)	39‐78	0.25‐4	> 4	2‐4	78	1	78	2

^a^ MIC, minimum inhibitory concentration.

^b^ MBC, minimum bactericidal concentration.

^c^ Col, colistin (used as a drug control). MIC tests were undertaken in duplicate and on two separate occasions.

—* = not determined; MBC/MIC: > 4 values for GaLM NPs indicate bacteriostatic activity. *P. aeruginosa* Xen41 bioluminescent bacteria is derived from parental strain *P. aeruginosa* PAO1.

### 3.3 GaLM NPs Show Activity Against Bacterial Pathogens in Combination With Other Antibiotics

GaLM NPs were assessed for synergy with vancomycin against Gram‐positive (MSSA and MRSA reference strains and isolates) and in combination with colistin against Gram‐negative (*P. aeruginosa* ATCC strains and clinical isolates). The results showed that GaLM NPs exhibited indifferent activity when combined with vancomycin and against the MSSA and MRSA reference strains and isolates (Table 3). However, GaLM NPs exhibited additive activity against two *P. aeruginosa* ATCC strains and clinical isolates when combined with sub‐inhibitory concentrations of colistin. GaLM NPs MIC range was 39–78 μg/mL; but when combined with sub‐inhibitory concentrations of colistin (0.125–1 μg/mL) the MIC range was 5–39 μg/mL. The MIC range of colistin alone was 0.25–2 μg/mL (Table 4, Supporting Information S1: Table S2 and Supporting Information S1: Figure S1).

**Table 3:** Indifferent activity of GaLM NPs + vancomycin combination against Gram‐positive bacterial strains.

*S. aureus* strains/isolates	MIC (µg/mL)^a^				FICI^b^	DRI^3^	
	Single antibiotic		Combination				
	Van	GaLM NPs	Van	GaLM NPs		Van	GaLM NPs
ATCC 29213 (MSSA)	1	78	1	78	2*	1	1
ATCC 49775 (MSSA)	1	78	1	78	2	1	1
USA300/SAP231 (MRSA)	1	156	1	156	2	1	1
ATCC 43300 (MRSA)	1	156	1	156	2	1	1
MW2/SAP227 (MRSA)	1	156	1	156	2	1	1

^a^ MIC, minimum inhibitory concentration.

^b^ FICI = Fractional inhibitory concentration index; synergistic (FICI ≤ 0.5); additive or partially synergistic (0.5 < FICI ≤ 1); indifferent (1 < FICI ≤ 4); and antagonistic (FICI > 4).

* = indifferent effect; ^3^DRI = Dose reduction index; Van, vancomycin.

**Table 4:** Additive activity of GaLM NPs + colistin combination against *P. aeruginosa* ATCC strains and clinical isolates.

*P. aeruginosa* strains/isolates	MIC (µg/mL)^a^				FICI	DRI	
	Single antibiotic		Combination				
	Col	GaLM NPs	Col	GaLM NPs		Col	GaLM NPs
PAO1 (Xen41)	0.5	78	0.25	10	0.63*	2	16
ATCC 27853	0.5	78	0.25	10	0.63*	2	16
Clinical isolates (*n* = 21)	0.25‐2	39‐78	0.03‐1	5‐39	0.56‐0.63*	2‐8	2‐16

^a^ MIC, minimum inhibitory concentration; FICI= Fractional inhibitory concentration index; synergistic (FICI ≤ 0.5); additive or partially synergistic (0.5 < FICI ≤ 1); indifferent (1 < FICI ≤ 4); and antagonistic (FICI > 4).

* = Additive effect; DRI = Dose reduction index; col, colistin.

### 3.4 GaLM NPs Exhibit Time‐ and Concentration‐Dependent Killing and Growth‐Inhibitory Activity against Gram‐Positive and Gram‐Negative Bacteria

**Figure 2:**
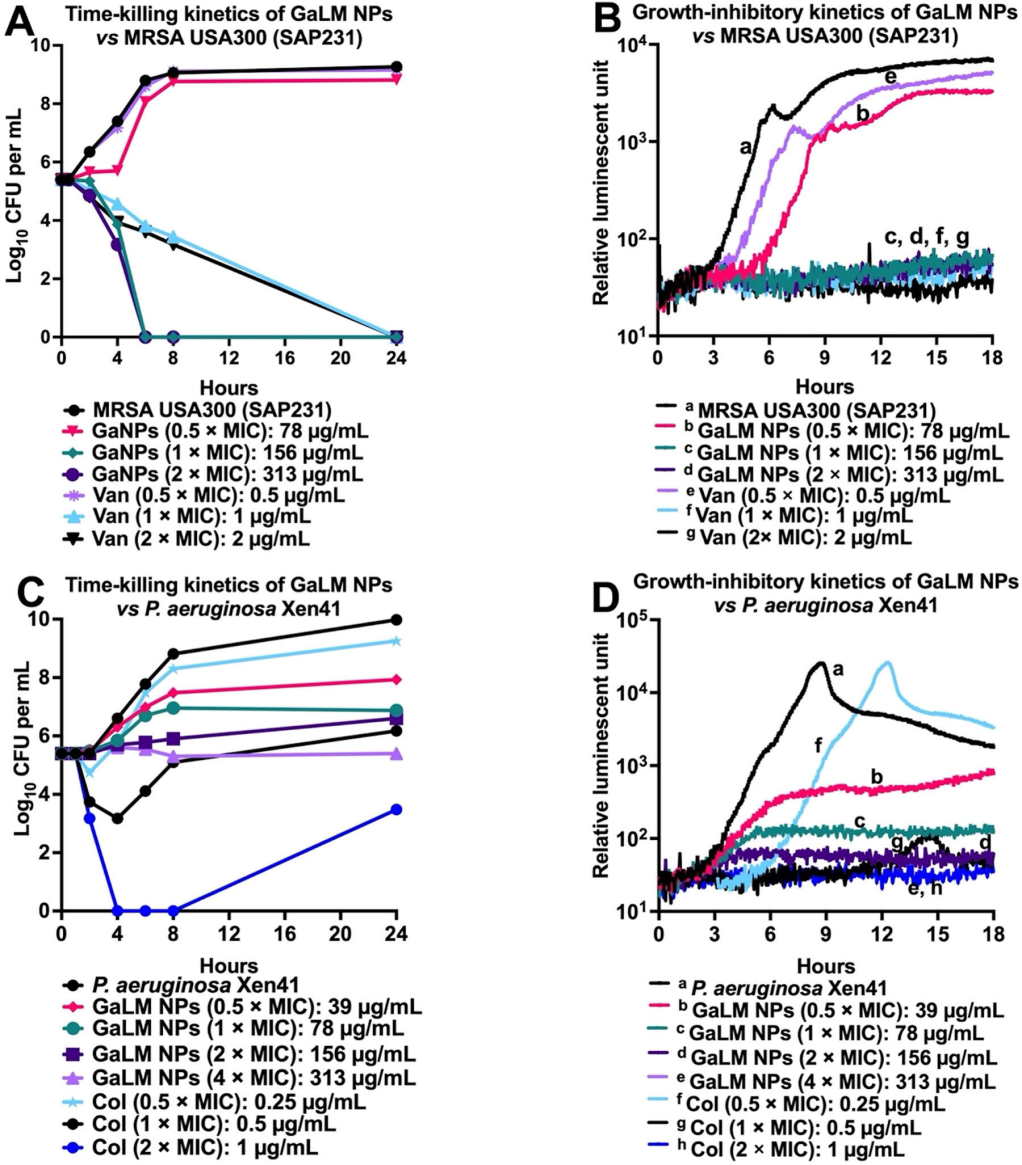
Time‐ and concentration‐ killing and growth inhibitory kinetics of GaLM NPs against Gram‐positive and Gram‐negative bacteria. GaLM NPs were prepared at 0.5 × MIC, 1 × MIC, 2 × MIC and 4 × MIC; vancomycin was prepared at 0.5 × MIC, 1 × MIC and 2 × MIC; colistin was prepared at 1 × MIC and 2 × MIC. (B and D), tests were prepared for MIC testing on clear, flat bottom black‐96‐well plates (CLS3603, Sigma‐Aldrich) for 18 h at 37°C on a Cytation 5 Cell Imaging Multi‐Mode Reader (BioTek). (A, C), tests were prepared in 5 mL yellow capped tubes; then samples were withdrawn at indicated times for serial dilutions and plating and incubation at 37°C overnight on HBA plates. Col, colistin; MIC, minimum inhibitory concentration; Van, vancomycin.

Time‐ and concentration‐dependent growth inhibition and killing of bacteria by GaLM NPs was assessed using bioluminescent MRSA USA300 (SAP231) (Figures 2A,B), bioluminescent *P. aeruginosa* Xen41 (Figures 2C,D) and bioluminescent *E. coli* Xen14 (Supporting Information S1: Figure S2). Bioluminescent MRSA USA300 (SAP231), *P. aeruginosa* PAO1 (Xen41) and *E. coli* WS2572 (Xen14) were used in this assay because optical density (*A*_600 nm_ measurements) could not be performed due to the black color of GaLM NPs, which causes increasing optical density (OD) values with increasing concentration of GaLM NPs (Supporting Information S1: S1). Therefore, the effects of GaLM NPs and control drugs on the growth of SAP231, Xen41 and Xen14 were determined via changes in the metabolic activity of the bacteria as measured by relative luminescence units (Figure 2B,D and Supporting Information S1: Figure S2).

GaLM NPs at 1 × MIC (156 μg/mL) killed MRSA USA300 (SAP231) faster than vancomycin at 1 × MIC (1 μg/mL) at 6 h posttreatment (Figure 2A), corresponding to the absence of metabolic activity of SAP231 treated with either GaLM NPs or vancomycin at their corresponding MICs (Figure 2B). The figure also shows the concentration‐dependent effects of GaLM NPs in reducing the metabolic activity of SAP231. Figure 2A,B shows a bactericidal pattern of GaLM NPs for SAP231, corroborating the results presented in Table 1. Figure 2C,D show time‐ and concentration‐dependent inhibitory of GaLM NPs against Xen41 and illustrate a bacteriostatic pattern. However, the GaLM NPs MIC was greater than 5000 μg/mL for Xen14 (Table 2). Interestingly, concentration‐dependent antimicrobial effects of GaLM NPs against Xen14 at concentrations ranging from 600 to 5000 μg/mL could be observed by metabolic activity measurements (Supporting Information S1: Figure S2).

### 3.5 Combinations (GaLM NPs + Vancomycin or GaLM NPs + Colistin) Exhibit Time‐ and Concentration‐Dependent Growth Inhibitory and Killing Kinetics Against MRSA USA300 (SAP231) and *P. aeruginosa* Xen41

**Figure 3:**
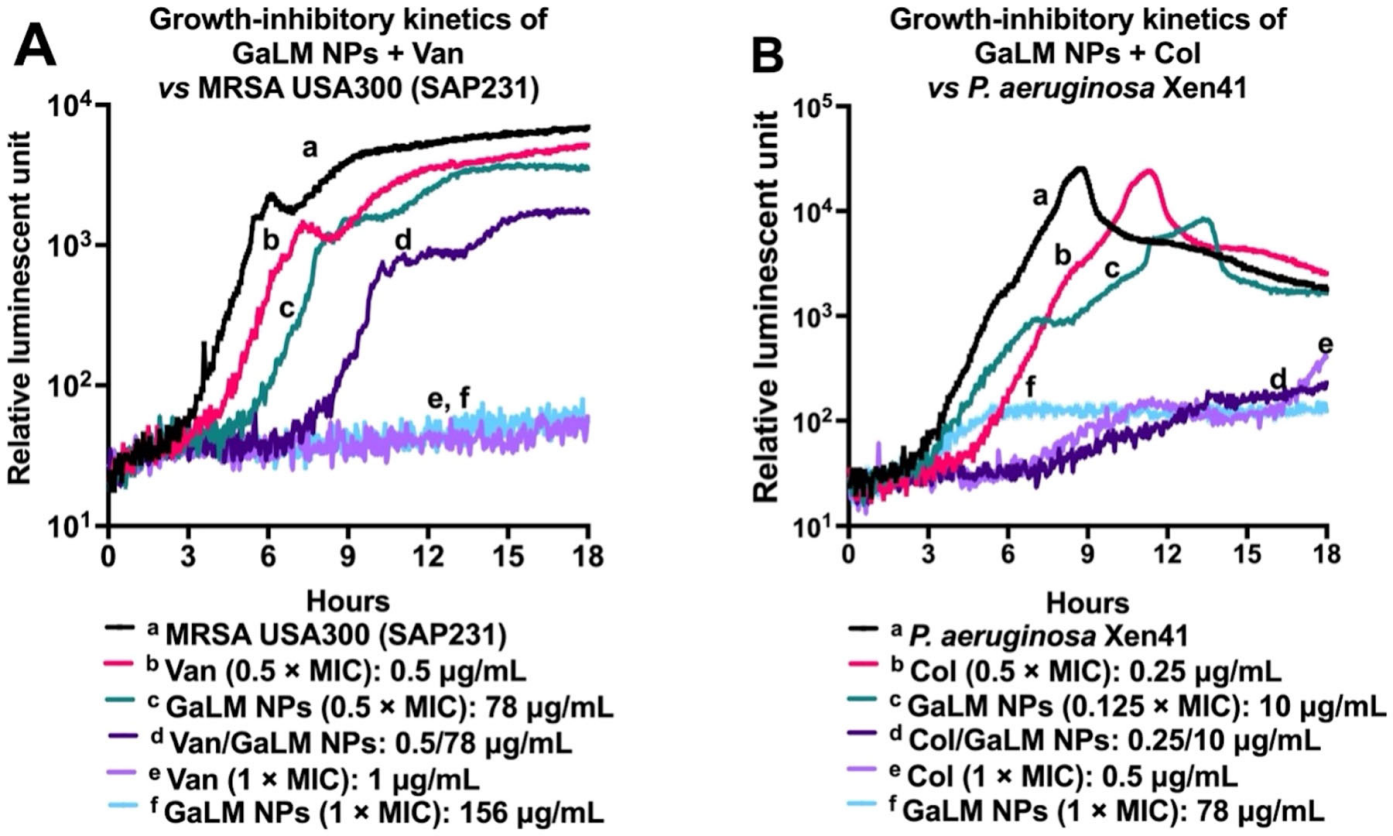
Time and concentration‐dependent growth inhibitory kinetics of GaLM NPs + vancomycin combination against Gram‐positive bacteria and GaLM NPs + colistin combination against Gram‐negative bacteria. The test was prepared as MIC on black‐96‐well plates with clear flat bottoms (CLS3603, Sigma‐Aldrich) for 18 h at 37°C on a Cytation 5 Cell Imaging Multi‐Mode Reader (BioTek). (A) time‐ and‐ concentration growth inhibitory kinetics of GaLM NPs + vancomycin combination against SAP231; (B) time‐and‐concentration growth inhibitory kinetics of GaLM NPs + colistin combination against Xen41. Col, colistin; MIC, minimum inhibitory concentration; Van, vancomycin.

Vancomycin/GaLM NPs combination (0.5/78 μg/mL) had no synergistic activity but delayed metabolic activity of SAP231 in comparison with vancomycin alone or GaLM NPs alone at the same concentrations (Figure 3A). Figure 3B further confirms the additive activity of GaLM NPs + colistin combination as indicated in Table 4. The colistin/GaLM NPs combination (0.25/10 μg/mL) caused a dramatic reduction in metabolic activity of Xen41 in comparison with GaLM NPs or colistin alone at the same concentrations.

### 3.6 Morphological Changes in Planktonic MRSA USA300 Cells After Treatment With Galm Nps

**Figure 4:**
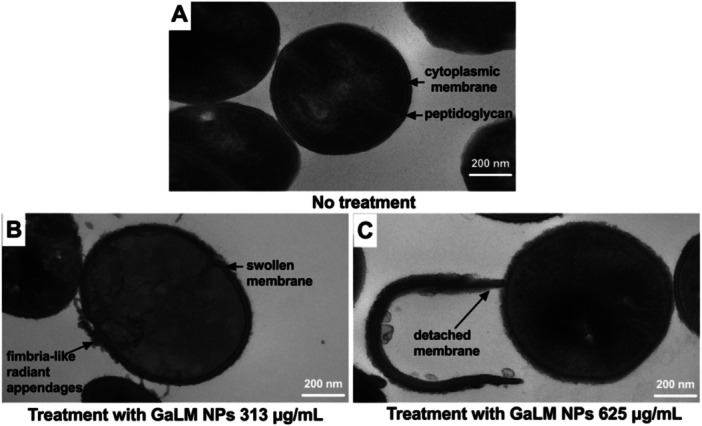
Concentration‐dependent effects of a 3 h GaLM NPs treatment on cellular morphology of MRSA USA300. (A) Bacterial cells without treatment showing intact cellular structure of peptidoglycan and cytoplasmic membrane; (B) cells treated with GaLM NPs at 313 μg/mL (2 × MIC) showing a swollen envelope with tubular and fimbria‐like radian appendages; (C) bacterial cells treated with GaLM NPs at 625 μg/mL (4 × MIC) showing detached and broken cell membrane; scale bar: 200 nm.

TEM images revealed that the cell envelope of untreated (control) MRSA USA300 was clearly visible and consisted of the plasma membrane and cell wall (Figure 4A). Cells treated with GaLM NPs at 313 μg/mL (2 × MIC) had a swollen envelope with fimbria‐like radiant appendages (Figure 4B). Increasing the concentration of GaLM NPs to 625 μg/mL (4 × MIC) led to greater cell damage resulting in broken and detached cell membranes (Figure 4C).

### 3.7 GaLM NPs Exhibit Antibiofilm Activity Against Gram‐Positive Bacteria

**Figure 5:**
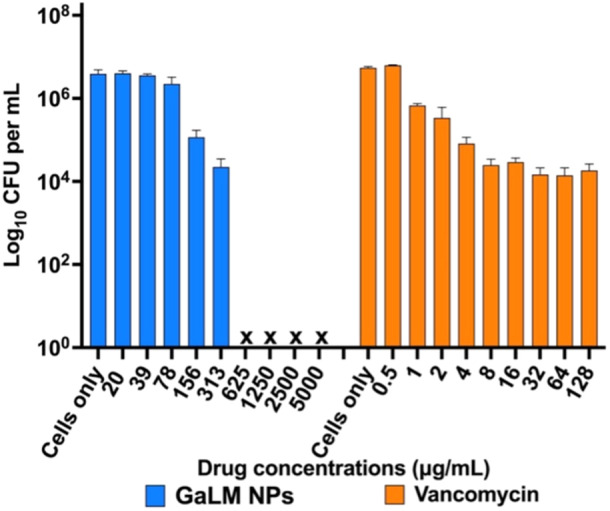
Biofilm activity of GaLM NPs and vancomycin against MRSA USA300. The graph shows the total number of viable cells remaining on the pegs of the MBEC lid after treatment with different concentrations of GaLM NPs and vancomycin. Data represents mean ± standard deviation (*n* = 6, two independent experiments).

**Figure 6:**
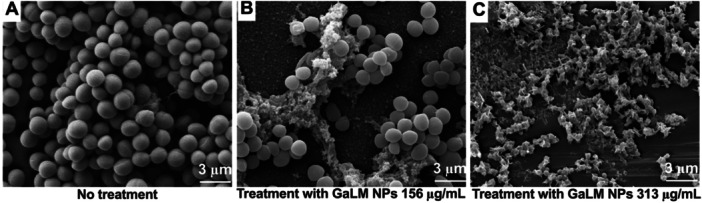
Representative SEM Images showing eradication of MRSA USA300 biofilms by GaLM NPs. (A) sample without treatment showing the bunched cell cluster and biofilm matrix; (B) sample treated with GaLM NPs at 156 μg/mL (1 × MIC) showing fewer cells compared to the untreated sample and changes in biofilm structure; (C) sample treated with GaLM NPs at 313 μg/mL (2 × MIC) showing eradication of more cells compared to 156 μg/mL treatment; scale bar: 3 μm.

When SAP231 was grown as a biofilm in the MBEC plate, GaLM NPs completely eradicated MRSA USA300 biofilms at a MBEC of 625 µg/mL (4 × MIC), whereas the MBEC of vancomycin was higher than 128 µg/mL (Table 5). Concentration‐dependent eradication of MRSA USA300 biofilms by GaLM NPs is presented in Figure 5 as a total number of viable cells remaining on pegs; also visualized via SEM imaging (Figure 6).

**Table 5:** Minimum biofilm eradication concentration for GaLM NPs against MRSA USA300 (SAP231).

Bacterial strain	MIC (µg/mL)^a^		MBEC (µg/mL)^b^	
	GaLM NPs	Van	GaLM NPs	Van
MRSA USA300	156	1	625	> 128

^a^ MIC: minimum inhibitory concentration.

^b^ MBEC: minimum biofilm inhibitory eradication concentration; Van, vancomycin. Biofilm susceptibility testing of GaLM NPs and vancomycin was tested in MBEC biofilm plates (MBEC Biofilm Inoculator, Innovotech, Edmonton, Canada). The tests were repeated on two independent occasions.

### 3.8 GaLM NPs Are Not Toxic to Human Epidermal Keratinocyte Cell Line

**Figure 7:**
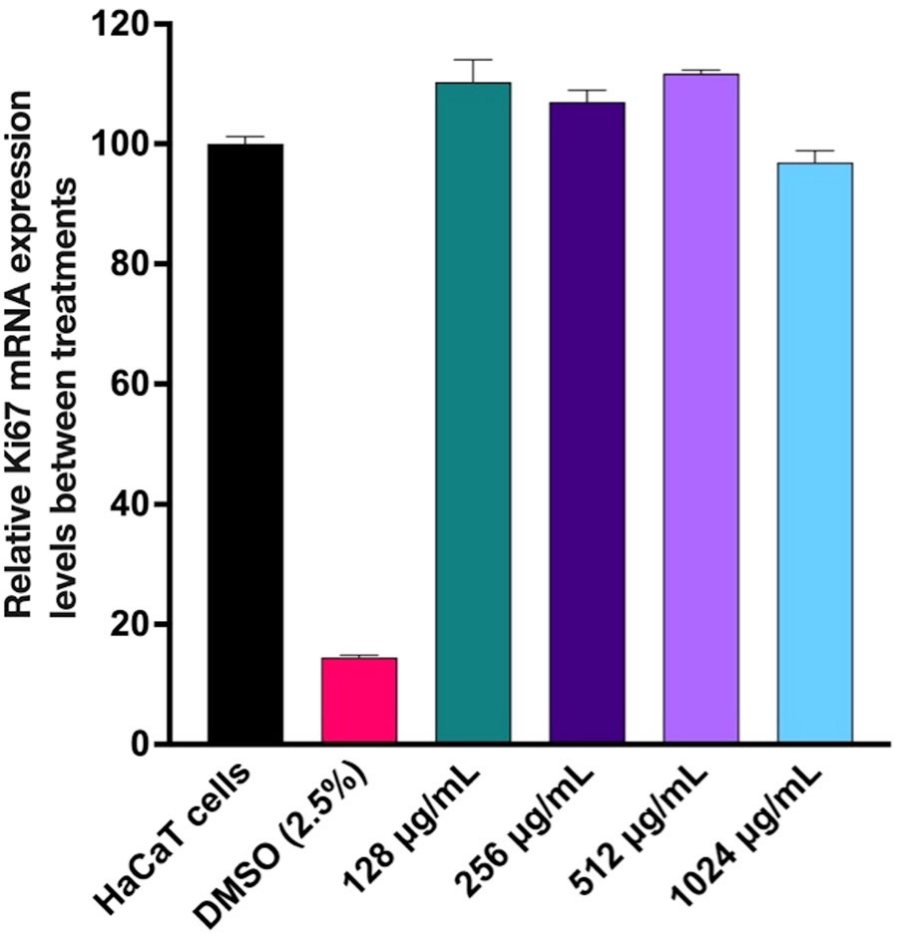
Cytotoxicity assessment of GaLM NPs on HaCaT cells. HaCaT cells were treated with different concentrations of GaLM NPs for 24 h using DMSO (2.5%) as a control for cytotoxicity. Untreated and treated cells were harvested to measure the expression of the proliferation marker gene Ki67 using quantitative real‐time PCR (TB Green Premix Ex Taq ‐Tli RNase H Plus, Takara Bio Inc). Data are means ± standard deviation (*n* = 3); DMSO, dimethyl sulfoxide.

The results of mammalian cell toxicity assays using human epidermal keratinocyte (HaCaT) cell line revealed that after 24 h of treatment, DMSO significantly inhibited cell proliferation by approx. 90%, while cells treated with different concentrations of GaLM NPs (ranging from 128 to 1024 µg/mL) showed a minimal impact on cell proliferation, with inhibition levels of less than 15%. This suggests that GaLM NPs treatment had a limited effect on the proliferation of HaCaT cells, particularly in comparison to the significant inhibition caused by dimethyl sulfoxide (DMSO) (Figure 7).

## 4 Discussion

Multidrug‐resistant pathogens continue to pose significant threats to healthcare and global public health as the efficacy of traditional antibiotics has been significantly reduced (O'Neill 2016). To address the growing challenge of bacterial resistance, various antimicrobial agents such as antimicrobial peptides, metals/metal oxides, quaternary ammonium salts, and graphene oxide, act through mechanisms including membrane disruption, oxidative stress, reactive oxygen species generation, and inhibition of microbial adhesion, have gained much attention (Hamad et al. 2020; Nie et al. 2023; Liu et al. 2025; Pereira et al. 2019). These metals have been applied in wound dressings, bone grafts, and tissue regeneration. Concerns remain regarding heavy metal toxicity due to difficulty in degrading in vivo and long‐term accumulation that may negatively impact health (Abd Elnabi et al. 2023). Ga has recently gained considerable attention due to its antibacterial properties and its safety has long been recognized (Truong et al. 2023; Liu et al. 2025; Frei et al. 2023). Ga^3+^ was approved by the FDA for the treatment of malignant tumor‐related hypercalcemia and autoimmune diseases in 2003 (Leyland‐Jones 2003). It was also reported that bacteria do not easily develop resistance to Ga^3+^ (Shi et al. 2023). Given the attractive properties of Ga, this study investigated the potential use of GaLM NPs as a novel technology for treating multidrug‐resistant pathogens.

We found that GaLM NPs showed bactericidal activity at MIC range of 39–156 μg/mL against Gram‐positive bacterial pathogens and bacteriostatic activity at MIC range of 39–625 μg/mL against Gram‐negative pathogens. TEM images show damage to the membrane of GaLM NPs‐treated MRSA USA300. GaLM NPs also eradicated MRSA USA300 biofilms at a MBEC of 625 μg/mL using an MBEC model, as demonstrated by SEM images. Together, these data indicate that GaLM NPs may possess physical and biological mechanisms to exert antimicrobial activity. Since the average size of the gaLM NPs was 401.2 ± 58.2 nm in diameter, the damage observed on the cells may be caused by the oxidative stress resulting from the strong interactions of GaLM NPs with the bacterial cell membrane, as previously discovered (Cheeseman et al. 2022a), rather than penetration into the cell.

The differences in the interaction of GaLM NPs between Gram‐positive and Gram‐negative bacteria in this study and reported previously (Cheeseman et al. 2022a) could be attributed to differences in bacterial membrane structure, with Gram‐negative bacteria being surrounded by a thin peptidoglycan (< 10 nm) and an additional outer membrane while Gram‐positive cells have thicker peptidoglycan (20‐80 nm) but lack an outer membrane (Silhavy et al. 2010). This may explain the bactericidal activity of GaLM NPs observed against *S. aureus* (MSSA and MRSA) isolates and bacteriostatic activity against Gram‐negative pathogens. Furthermore, the larger GaLM NPs may be primarily responsible for physically disrupting the biofilm matrix, while the smaller GaLM NPs can embed themselves within the bacterial cell membrane, leading to the killing of planktonic cells and eradication of MRSA USA300 biofilms. Furthermore, GaLM NPs may rely on a unique “Trojan horse”‐type mechanism, whereby Ga may be oxidized to Ga_2_O_3_, which reacts with water to form GaOOH at room temperature (Shi et al. 2023), and then Ga^3+^ can act as an iron mimic to infiltrate into the bacterial cytoplasm to exert antimicrobial activity (Goss et al. 2018). This hypothesis will be further examined in future research by using synthetic chelators of Ga^3+^ before testing the effect of gallium against bacteria. Notably, we observed that GaLM NPs at 1 × MIC (156 μg/mL) killed MRSA USA300 faster than the membrane‐active vancomycin at 1 × MIC (1 μg/mL) in two independent experiments, suggesting that the antimicrobial activity of GaLM NPs might be due to targeting of multiple targets at the cell surface.

The potential of combining Ga in ion forms with marketed antibiotics against bacterial pathogens was explored, such as the synergistic activity of Ga^+^ with colistin/polymyxin B against pan‐resistant *A. baumannii* and polymyxin‐resistant *K. pneumoniae* strains (Antunes et al. 2012; Rossato et al. 2022). We found additive activity when combining GaLM NPs with sub‐inhibitory concentrations of colistin against a wide range of clinical *P. aeruginosa* isolates using checkerboard assays and growth‐inhibitory kinetics. Colistin is considered the last resort antibiotic class for the treatment of Gram‐negative bacterial infections (Bergen et al. 2015; Bush 2015) due to its target site being the outer membrane, which prevents drugs from inserting into the inner membrane to kill bacteria. However, colistin is associated with nephrotoxicity, neurotoxicity and neuromuscular blockade (Justo and Bosso 2015; Nigam et al. 2015), limiting its use in clinical settings. Therefore, the findings in this study add value to several studies that support a new strategy of combining low concentrations of polymyxins (polymyxin B and colistin) with other antibiotics or nonantibiotic agents, adjuvants and antibiotic resistance breakers to treat Gram‐negative infections (Antunes et al. 2012; Nguyen et al. 2021; Rossato et al. 2022; Guo et al. 2023). Also, we found that while combination of GaLM NPs and vancomycin exhibited no synergistic activity against MRSA reference strains, it delayed metabolic activity of the cells. This might allow the host's immune system to eliminate the bacteria, provide adequate duration for drug therapy and/or reduce the risk of resistance and preserve beneficial bacteria during therapy.

Inplantable device‐related infections are one of the most severe complications in surgery (Moriarty et al. 2016). The infections result in significantly reduced clinical outcomes, such as increased postoperative pains, mortality, delayed functional impairment, prolonged recovery time and higher hospitalization costs (Gan 2017). Perioperative antibiotic prophylaxis is a common practice to decrease the incidence of postoperative infections (Prokuski 2008). However, the use of high antimicrobial doses to reach surgical sites due to impaired vascularization resulting from the initial trauma and to halt bacterial biofilm formation is associated with systemic toxicity and side effects (Hrynyshyn et al. 2022). Developing GaLM NPs coatings for preventing or treating medical device‐related infections could be a potent strategy forward. In the current study, we found that GaLM NPs can eradicate MRSA biofilms along with enhancing the antimicrobial activity of other drugs (vancomycin and colistin) and also demonstrated the in vitro safety of GaLM NPs at 1024 μg/mL which is higher than its MIC against the bacteria tested. These findings provide essential data for preclinical studies to test the efficacy of GaLM NPs‐coated implants in mouse models of bacterial infections as proof of concept.

## 5 Conclusion

The results of this study support the potential of GaLM NPs as a nonantibiotic‐dependent antimicrobial strategy to prevent or treat multidrug Gram‐positive or Gram‐negative infections. In addition, GaLM NPs can enhance the efficacy of conventional antibiotics such as vancomycin and colistin, offering new avenues to reduce drug toxicity and minimize the emergence of resistance. Importantly, due to the potentially multiple mechanisms of GaLM NPs, this suggests a reduced likelihood that pathogens will develop resistance to GaLM NPs. Therefore, GaLM NPs are promising as an alternative to other metals, such as silver nanoparticles, to which rapid development of resistance among clinical isolates has been reported (Graves et al. 2015). This study further confirmed the low cytotoxicity of GaLM NPs. These findings indicate that GaLM NPs represent a next‐generation antimicrobial platform with potential for translational application. Therefore, preclinical in vivo efficacy and toxicity studies are warranted to further delineate their pharmacokinetic properties, therapeutic index, and long‐term biosafety profile.

## Author Contributions

**Hang Thi Nguyen:** conceptualization, data curation, formal analysis, investigation and methodology, validation, visualization, writing – original draft, writing – review and editing. **Jiratchaya Puangseree:** data curation, formal analysis, investigation and methodology, validation, visualization, writing – original draft, writing – review and editing. **Tien Thanh Nguyen:** data curation, formal analysis, investigation and methodology, validation, visualization, writing – original draft, writing – review and editing. **Manh Tuong Nguyen:** data curation, formal analysis, investigation and methodology, validation, visualization, writing – original draft, writing – review and editing. **Christopher Leigh:** data curation, formal analysis, investigation and methodology, validation, visualization, writing – original draft, writing – review and editing. **Henrietta Venter:** formal analysis, resources, supervision, writing – review and editing. **Krasimir Vasilev:** conceptualization, resources, supervision, validation, writing – review and editing. **Rungtip Chuanchuen:** resources, supervision, writing – review and editing. **Darren J. Trott:** resources, supervision, writing – review and editing. **Vi Khanh Truong:** conceptualization, formal analysis, resources, supervision, validation, writing – review and editing. **Abiodun David Ogunniyi:** conceptualization, data curation, formal analysis, resources, supervision, validation, visualization, writing – review and editing. All authors read and approved the submitted version of the manuscript, in addition to contributing to manuscript revision.

## Ethics Statement

The authors have nothing to report.

## Conflicts of Interest

The authors declare no conflicts of interest.

## Supporting information


**Figure S1:** A representative checkerboard plate shows the synergy of GaLM NPs + colistin combination against P. aeruginosa cnl17. **Figure S2:** Time and concentration‐dependent growth inhibitory kinetics of GaLM NPs against E. coli Xen14. **Table S1:** GaLM NPs show activity against S. aureus reference strains and MRSA clinical isolates. **Table S2:** Activity of GaLM NPs against P. aeruginosa reference strains and clinical isolates.

## Data Availability

All data generated or analyzed during this study have been provided within the published article or through supporting data files.
