# Supplementary material for: Gallium Liquid Metal Nanoparticles as Agents to Treat Multidrug‐Resistant Bacterial Infections
Source: Microbiologyopen. 2025 Oct 23;14(5):e70078. doi: 10.1002/mbo3.70078 (PMC12548495; doi:10.1002/mbo3.70078)
Supplement: Supplementary file 1 — Figure S1: A representative checkerboard plate shows the synergy of GaLM NPs + colistin combination against P. aeruginosa cnl17. Figure S2: Time and concentration‐dependent growth inhibitory kinetics of GaLM NPs against E. coli Xen14. Table S1: GaLM NPs show activity against S. aureus reference strains and MRSA clinical isolates. Table S2: Activity of GaLM NPs against P. aeruginosa reference strains and clinical isolates. [file MBO3-14-e70078-s001.pdf]

**SUPPLEMENTARY RESULTS**

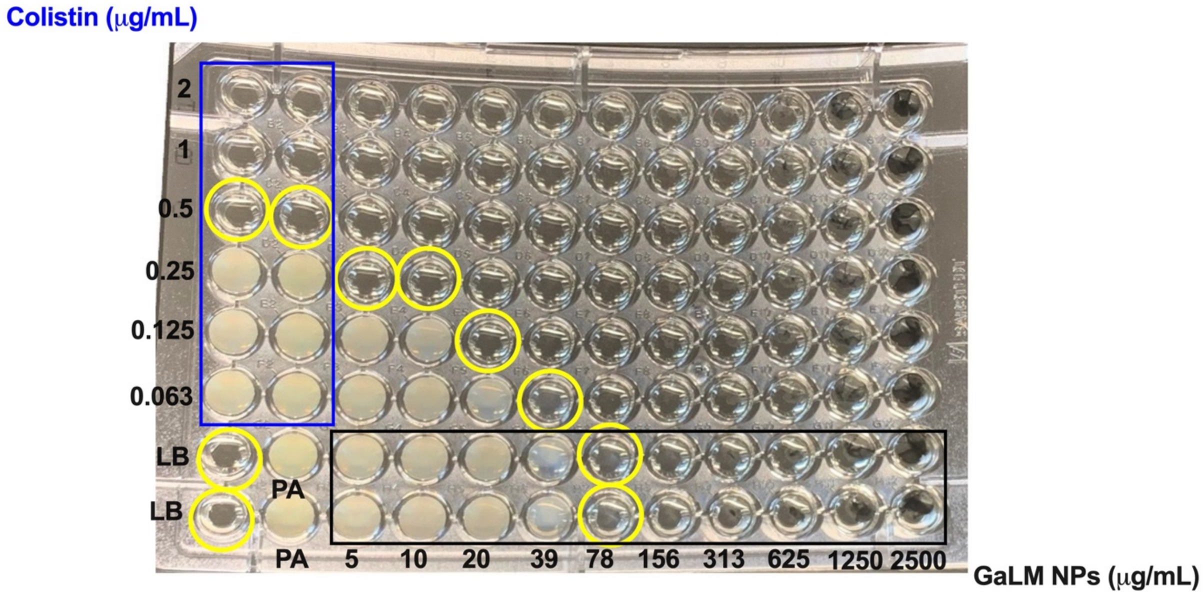


**Figure S1. A representative checkerboard plate shows the synergy of GaLM NPs + colistin combination against *P. aeruginosa* cnl17.** Colistin concentrations ranged from 0.063 to 2 μg/mL (row A to F); GaLM NPs concentrations ranged from 5 μg/mL to 2500 μg/mL (columns 3-12); black box, GaLM NPs alone; blue box, colistin alone, all other wells, combination of colistin and GaLM NPs; PA, *P. aeruginosa*, bacterial growth control containing only *P. aeruginosa* cnl17; LB, LB broth only (no growth control, also as a base to determine the MIC of colistin alone, GaLM NPs alone or colistin + GaLM NPs combination); yellow circles indicated broth only and MIC of each drug and combinations.

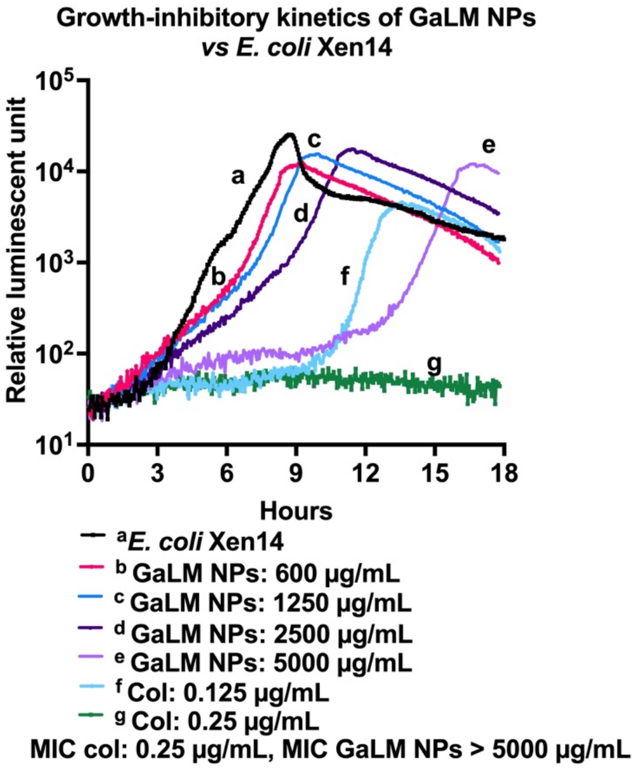


**Figure S2**. **Time and concentration-dependent growth inhibitory kinetics of GaLM NPs against *E. coli* Xen14**. The test was prepared as MIC on black-96-well plates with clear flat bottoms (CLS3603, Sigma-Aldrich) for 18 h at 37 °C on a Cytation 5 Cell Imaging Multi-Mode Reader (BioTek). MIC, minimum inhibitory concentration; Col, colistin.

**Table S1. GaLM NPs show activity against *S. aureus* reference strains and MRSA clinical isolates**

| Number | *S. aureus* strains/isolates | MIC (µg/mL) | | MBC (µg/mL) | |
|---|---|---|---|---|---|
| | | GaLM NPs | Van | GaLM NPs | Van |
| 1 | MRSA MW2/SAP227 | 156 | 1 | 156 | 1 |
| 2 | MRSA USA300/SAP231 | 156 | 1 | 156 | 1 |
| 3 | MRSA ST398 | 156 | 1 | 156 | 1 |
| 4 | MRSA WSPP | 78 | 1 | 156 | 1 |
| 5 | MRSA NYJapan | 156 | 1 | 156 | 1 |
| 6 | MRSA TAI PVL+ | 156 | 1 | 156 | 1 |
| 7 | MRSA UK17 | 156 | 1 | 156 | 1 |
| 8 | MRSA UK16 | 156 | 1 | 156 | 1 |
| 9 | MRSA UK15 | 156 | 1 | 156 | 1 |
| 10 | MRSA PVL+ | 78 | 1 | 78 | 1 |
| 11 | MRSA QLD PVL+ | 156 | 1 | 156 | 1 |
| 12 | MRSA WA1 | 156 | 1 | 156 | 1 |
| 13 | MRSA WA2 | 156 | 1 | 156 | 1 |
| 14 | MRSA AUS3 | 78 | 1 | 78 | 1 |
| 15 | MRSA AUS2 | 78 | 1 | 78 | 1 |
| 16 | MRSA Bendgal Bay PVL+ | 156 | 1 | 156 | 1 |
| 17 | MRSA Classic | 156 | 1 | 156 | 1 |
| 18 | MRSA Irish 2 | 78 | 1 | 78 | 1 |
| 19 | MRSA Irish 1 | 78 | 1 | 78 | 1 |

| | | | | | |
|---|---|---|---|---|---|
| 20 | MRSA WA3 | 156 | 1 | 156 | 1 |
| **MIC/MBC Range** | | **78-156** | **1** | **39-156** | **1** |
| VISA (ATCC 700699) | | 39 | 4 | 39 | 4 |
| ATCC (Xen29) | | 78 | 1 | 78 | 1 |
| ATCC 49775 | | 78 | 1 | 78 | 1 |
| ATCC 29213 | | 78 | 1 | 78 | 1 |

**Table S2. Activity of GaLM NPs against *P. aeruginosa* reference strains and clinical isolates**

| Number | *P. aeruginosa* strains/isolates | MIC (µg/mL) | | MBC/MIC | |
|---|---|---|---|---|---|
| | | GaLM NPs | Col | GaLM NPs | Col |
| 1 | cln 1 | 78 | 1 | > 4 | 2 |
| 2 | cln 2 | 39 | 0.25 | > 4 | 2 |
| 3 | cln 3 | 78 | 0.5 | > 4 | 2 |
| 4 | cln 4 | 78 | 0.25 | > 4 | 2 |
| 5 | cln 5 | 78 | 2 | > 4 | 2 |
| 6 | cln 6 | 39 | 2 | > 4 | 2 |
| 7 | cln 7 | 39 | 1 | > 4 | 2 |
| 8 | cln 8 | 78 | 1 | > 4 | 2 |
| 9 | cln 9 | 78 | 2 | > 4 | 2 |
| 10 | cln 10 | 78 | 2 | > 4 | 2 |
| 11 | cln 11 | 39 | 2 | > 4 | 2 |
| 12 | cln 12 | 78 | 2 | > 4 | 2 |
| 13 | cln 13 | 39 | 2 | > 4 | 2 |
| 14 | cln 14 | 39 | 2 | > 4 | 2 |
| 15 | cln 15 | 78 | 0.5 | > 4 | 2 |
| 16 | cln 16 | 78 | 0.25 | > 4 | 2 |
| 17 | cln 17 | 78 | 0.25 | > 4 | 2 |
| 19 | cln 18 | 39 | 0.25 | > 4 | 2 |
| 19 | cln 19 | 39 | 1 | > 4 | 2 |

| | | | | | |
|---|---|---|---|---|---|
| 20 | cln 20 | 78 | 0.5 | > 4 | 2 |
| 21 | cln 21 | 39 | 0.5 | > 4 | 2 |
| **MIC/ MBC range** | | **39-78** | **0.25-2** | **> 4** | **2** |
| PAO1/Xen41 | | 78 | 0.5 | > 4 | 2 |
| ATCC 27823 | | 78 | 0.5 | > 4 | 2 |
